# Osteocyte *Egln1*/Phd2 links oxygen sensing and biomineralization via FGF23

**DOI:** 10.1038/s41413-022-00241-w

**Published:** 2023-01-18

**Authors:** Megan L. Noonan, Pu Ni, Emmanuel Solis, Yamil G. Marambio, Rafiou Agoro, Xiaona Chu, Yue Wang, Hongyu Gao, Xiaoling Xuei, Erica L. Clinkenbeard, Guanglong Jiang, Sheng Liu, Steve Stegen, Geert Carmeliet, William R. Thompson, Yunlong Liu, Jun Wan, Kenneth E. White

**Affiliations:** 1 Department of Medical and Molecular Genetics, Indiana University School of Medicine, Indianapolis, IN 46202 USA; 2 Laboratory of Clinical and Experimental Endocrinology, Department of Chronic Diseases and Metabolism, KU Leuven, 3000 Leuven, Belgium; 3 Department of Physical Therapy, Indiana University School of Medicine, Indianapolis, IN 46202 USA; 4 Department of Anatomy, Cell Biology, and Physiology, Indiana University School of Medicine, Indianapolis, IN 46202 USA; 5 Center for Computational Biology and Bioinformatics, Indiana University School of Medicine, Indianapolis, IN 46202 USA; 6 Departments of Medicine/Division of Nephrology, Indiana University School of Medicine, Indianapolis, IN 46202 USA

**Keywords:** Bone, Metabolic bone disease

## Abstract

Osteocytes act within a hypoxic environment to control key steps in bone formation. FGF23, a critical phosphate-regulating hormone, is stimulated by low oxygen/iron in acute and chronic diseases, however the molecular mechanisms directing this process remain unclear. Our goal was to identify the osteocyte factors responsible for FGF23 production driven by changes in oxygen/iron utilization. Hypoxia-inducible factor-prolyl hydroxylase inhibitors (HIF-PHI) which stabilize HIF transcription factors, increased Fgf23 in normal mice, as well as in osteocyte-like cells; in mice with conditional osteocyte *Fgf23* deletion, circulating iFGF23 was suppressed. An inducible MSC cell line (‘MPC2’) underwent FG-4592 treatment and ATACseq/RNAseq, and demonstrated that differentiated osteocytes significantly increased HIF genomic accessibility versus progenitor cells. Integrative genomics also revealed increased prolyl hydroxylase *Egln1* (Phd2) chromatin accessibility and expression, which was positively associated with osteocyte differentiation. In mice with chronic kidney disease (CKD), Phd1-3 enzymes were suppressed, consistent with FGF23 upregulation in this model. Conditional loss of Phd2 from osteocytes in vivo resulted in upregulated Fgf23, in line with our findings that the MPC2 cell line lacking Phd2 (CRISPR Phd2-KO cells) constitutively activated Fgf23 that was abolished by HIF1α blockade. In vitro, Phd2-KO cells lost iron-mediated suppression of Fgf23 and this activity was not compensated for by Phd1 or −3. In sum, osteocytes become adapted to oxygen/iron sensing during differentiation and are directly sensitive to bioavailable iron. Further, Phd2 is a critical mediator of osteocyte FGF23 production, thus our collective studies may provide new therapeutic targets for skeletal diseases involving disturbed oxygen/iron sensing.

## Introduction

Maintenance of normal mineral balance is necessary for proper structure of the skeleton, which also provides the long-term provision of phosphate and calcium as substrates for cellular use. Fibroblast growth factor-23 (FGF23) is a key factor involved in the endocrine axis regulating mineral ion handling. In this regard, FGF23 is secreted from bone cells (osteoblasts and osteocytes) in response to elevated circulating levels of phosphate or 1,25-dihydroxyvitamin D (1,25D). In the kidney, FGF23 regulates serum phosphate by reducing proximal tubule sodium-phosphate transporter content via downstream signaling initiated by binding to FGF receptors (FGFRs) and its co-receptor αKlotho.^1^ These interactions reduce blood 1,25D through downregulation of the 1,25D anabolic 1α-hydroxylase enzyme (Cyp27b1) and upregulation of the catabolic 24-hydroxylase (Cyp24a1).^2^ In addition to factors involved in mineral metabolism, other regulators of FGF23 production have been identified that tie systemic oxygenation to bone, including hypoxia and iron deficiency anemia.^3,4^ Osteocytes, which comprise over 95% of bone cells, reside in a hypoxic environment, with oxygen tensions below 10% (cortical bone pO_2_ of ~30 mmHg).^5,6^ Some studies suggest oxygen concentrations may be even lower as osteocytes are deeply embedded in matrix and have little to no direct connection to blood flow.^7^ The bone marrow may also contribute to the low oxygen tension in osteocytes since they consume much of the oxygen within the tissue.^8^ Importantly, hypoxia mediates the maturation of osteoblast into osteocytes,^9^ as well as directly regulates genes responsible for Wnt-mediated bone formation.^10^ Despite this, there is still conflicting evidence as to the role of hypoxia in osteocytes to control bone remodeling, and less is known as to its potential crossover roles in mineral homeostasis.

Importantly, iron deficiency anemia (IDA) and hypoxia stimulate the body’s primary oxygen adapters, hypoxia-inducible factors (HIFs). The HIF transcription factors are continuously turned over within cells as on-demand oxygen and iron sensors through the actions of the HIF-prolyl hydroxylase (HIF-PHD) family of enzymes. With inhibition or targeted loss of HIF-PHDs, HIFs are not recognized by von-Hippel Lindau (VHL), an E3 ubiquitin ligase that tags proteins for degradation by the proteasome. Under these conditions, HIFs remain intact and modify gene expression required to balance oxygen use, including stimulating primary factors Erythropoietin (EPO) and vascular endothelial growth factor (VEGF).^11,12^ The crossover regulation of the systemic control of phosphate and oxygen/iron homeostasis was derived as a key finding from the studies of a mouse model and patients with late-onset autosomal dominant hypophosphatemic rickets (ADHR).^3,13,14^ ADHR is caused by gain-of-function mutations in the *FGF23* gene that impair protease cleavage and inactivation, leading to increased blood bioactive intact ‘iFGF23’, hypophosphatemia secondary to renal phosphate wasting, causing markedly reduced bone mineralization and rickets/osteomalacia.^15^ These manifestations arise in patients during times of anemia or iron deficiency (i.e., pregnancy, puberty). To test the hypothesis that iron deficiency anemia was driving this phenotype, ADHR mice were fed a low-iron diet to induce iron deficiency anemia.^3^ The ADHR mice receiving the low iron diet increased iFGF23, whereas wild type (WT) mice retained the ability to cleave FGF23 protein into inactive fragments. This study demonstrated the link between iron and oxygen sensing pathways in osteocytes regulating FGF23, and is in line with previous findings of HIF activation of FGF23 transcription.^3,4,16,17^ Consistent with the pre-clinical ADHR mouse findings, patients with ADHR have elevated intact, bioactive FGF23.^18^ These patients can be cured with iron treatment to suppress FGF23,^19^ however, the mechanisms controlling FGF23 during hypoxia/anemia, as well as the direct actions of iron therapy on osteocytes are unknown.

Chronic kidney disease (CKD) is a very common disorder and is characterized by reduced expression of the FGF23 co-receptor αKlotho.^20–23^ This shift leads to increased FGF23 and drives the suppression of 1,25D and secondary hyperparathyroidism in CKD, causing severe bone disease. A critical manifestation arising in CKD is anemia due to lack of EPO production and/or EPO resistance, inflammation, or bleeding.^24^ Delivery of a novel class of oxygen mimetics, hypoxia-inducible factor-prolyl hydroxylase inhibitors (HIF-PHI) stabilize HIFs via PHD inhibition which increase endogenous EPO, or recombinant EPO itself, can cure the anemia of CKD.^25,26^ With resolving the IDA of a CKD mouse model, it was shown that FGF23 levels are reduced, supporting that FGF23 is strongly stimulated by hypoxia/anemia in this disease. Recent studies demonstrate that the cardiac hypertrophy often observed in CKD patients is associated with FGF23 effects via Klotho-independent mechanisms in heart.^27,28^ In the Chronic Renal Insufficiency Cohort (CRIC) trial, it was shown that heart failure and mortality is primarily driven by anemia-dependent FGF23 production.^29^ Collectively, these findings underscore that FGF23 production in osteocytes is highly responsive to hypoxia/anemia, which may lead to disease manifestations. Although these findings suggest common mechanisms between the regulation of mineral metabolism and osteocyte oxygen responses, the key cellular sensing mechanisms controlling bone FGF23 production during anemia remain poorly defined.

Herein, we identified Egl-9 Family Hypoxia Inducible Factor 1 (*Egln1*; PHD2) to be highly elevated in osteocytes in response to changes in oxygen sensing. We found that this enzyme is a critical mediator of FGF23 production using CRISPR-mediated targeting in a novel mesenchymal stem cell line (‘MPC2’ cells) in vitro and conditional deletion of *Egln1* from osteocytes in vivo that resulted in enhanced basal FGF23 production. Further, osteocytes directly controlled HIF-dependent FGF23 production via interactions with holo-transferrin during mimicked iron deficiency. Unbiased RNAseq/ATACseq determined that osteocytes rapidly respond to changes in oxygen/iron utilization through alterations in HIF-sensitive genomic accessibility that drive transcriptional reprogramming of adaptive pathways. Collectively, our results demonstrate that osteocytes are adapted to respond to changes in oxygen and iron via genomic and transcriptional mechanisms, and that osteocyte-expressed Phd2 is a critical sensor for FGF23 responsiveness. These findings provide critical junctures for understanding diseases with parallel disturbed oxygen/iron use and defective FGF23-driven mineral metabolism.

## Results

### Mimicked hypoxia induces FGF23 in bone cells directly in vitro, and in vivo

**Fig. 1:**
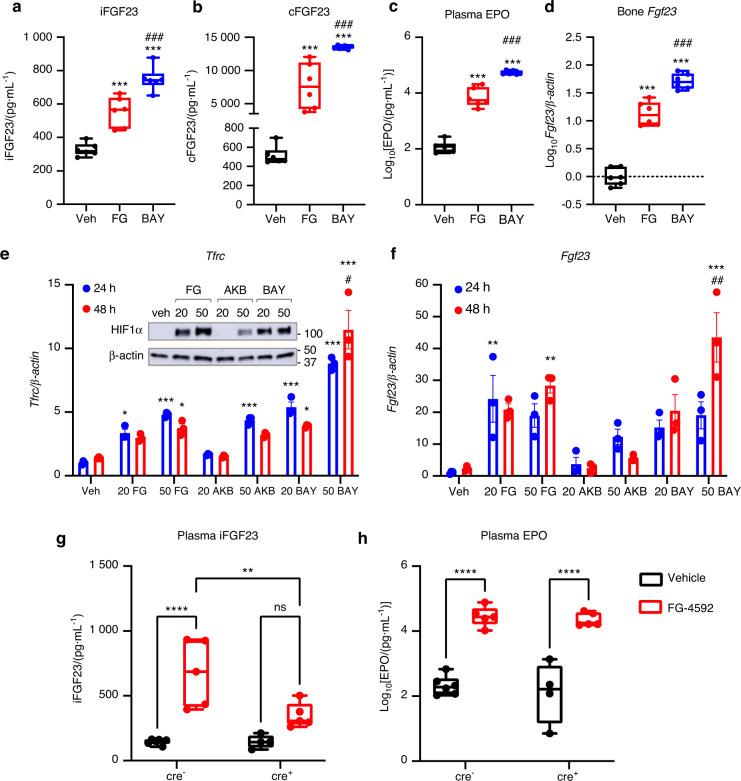
HIF-PHI induce FGF23 in WT mice and murine osteocyte-like cells. Wild type mice (females) were injected with vehicle (PEG300/DMSO) or 50 mg·kg^−1^ of either FG-4592 (‘FG’, Roxadustat; red) or BAY 85-3934 (‘BAY’, Molidustat; blue) every other day for 5 days, for a total of 3 injections. **a** Plasma intact FGF23 and **b** *C*-terminal/total FGF23 concentrations at 4 hours after final injection. **c** Plasma erythropoietin (EPO) concentrations and **d** cortical bone (flushed of marrow) *Fgf23* mRNA expression at the end of the study (*n* = 6 mice per group; ***P < 0.001 versus vehicle, ^###^*P* < 0.001 versus FG treatment). **e** Transferrin receptor (*Tfrc*) and **f** *Fgf23* mRNA expression in 2-week differentiated MPC2 cells treated for 24 h (blue) or 48 h (red) with 20 μmol·L^−1^ or 50 μmol·L^−1^ of the HIF-PHI FG-4592 (‘FG’; Roxadustat), AKB-6548 (‘AKB’; Vadadustat), or BAY 85-3934 (‘BAY’; Molidustat) (*P < 0.05, **P < 0.01, ***P < 0.001 versus vehicle; ^#^*P* < 0.05, ^##^*P* < 0.01 24 h versus 48 h). (**e**, inset) HIF1α protein expression in MPC2 cells after 4 h of 20 μmol·L^−1^ or 50 μmol·L^−1^ HIF-PHI treatment. Flox-*Fgf23*/*Dmp1*-cre^+^ and cre^−^ mice were injected with vehicle (black) or 70 mg·kg^−1^ FG-4592 (red) every other day for 5 days, for a total of 3 injections. Plasma concentrations of **g** intact FGF23 and **h** EPO were measured 4 hours after final injection (*n* = 4–6 mice per group; **P < 0.01, ****P < 0.000 1)

Osteocytes are central for mediating paracrine/endocrine homeostatic processes that maintain skeletal mass and biomineralization. It is known that these cells exist in a hypoxic milieu, and thus may be differentially sensitive to oxygen levels, however, the mechanisms that bridge oxygen sensing and bone function remain unresolved. During our studies of HIF-PHI treatment in CKD mice,^25,26^ and consistent with work of others,^4^ we discovered that the HIF-PHIs FG-4592 (Roxadustat) and BAY85-3934 (Molidustat) increased FGF23 in wild type (WT) mice. Due to the critical nature of the hypoxic environment of osteocytes in the regulation of FGF23, we used these analogs as biological tools to test the relationships between oxygen sensing and biomineralization through FGF23. To test osteocyte responses, 8-week-old female C57BL/6 WT mice were injected with 50 mg·kg^−1^ of FG-4592 or BAY85-3934 every other day for 5 days for a total of 3 injections, and tissues and blood were collected 4 hours after the final injection. At the time of harvest, plasma bioactive intact FGF23 (‘iFGF23’) was significantly increased in vivo following drug treatment (Fig. 1a). This response was also present as detected in an ELISA that measures both *C*-terminal fragments and full-length FGF23 (‘cFGF23’ or ‘total’ FGF23; Fig. 1b), supporting the association of HIF activation and FGF23 production. Plasma EPO levels were markedly elevated with FG-4592 and BAY85-3934 treatment compared to vehicle, and BAY85-3934 induced significantly higher levels of EPO compared to FG-4592 at the same dose (Fig. 1c), indicating BAY85-3934 is more potent in this regard. Cortical bone expression of Fgf23 mRNA was also increased following both FG-4592 or BAY85-3934 treatment (Fig. 1d). Thus, these findings support that oxygen sensing pathways influence the production and forms of secreted FGF23.

To investigate whether oxygen sensing pathways directly regulate FGF23 expression in bone cells, and to bridge mechanistic insight with the in vivo studies above, two cell lines were tested, a novel conditionally-immortalized mesenchymal progenitor cell line, clone 2 (‘MPC2’)^30^ and the human U2OS osteoblastic cell line. The advantage of the MPC2 cell line over primary mesenchymal stem cells (MSCs) is that MPC2s are temperature sensitive cells, proliferating at 33 °C. When cultured at 37 °C and supplemented with osteogenic media, MPC2 cells cease dividing and differentiate into osteocytes. To test for FGF23 responsiveness to mimicked hypoxia, MPC2 and U2OS cells were treated with 2 doses of 3 different clinical HIF-PHIs, FG-4592, BAY85-3934, and AKB-6548 (Vadadustat; ‘AKB’). In 2-week differentiated MPC2 cells, FG-4592 and BAY induced the HIF-responsive gene Transferrin receptor (Tfrc) mRNA expression, whereas AKB only induced expression using the higher dose after 24 hours (Fig. 1e). Both doses of FG-4592 or BAY85-3934 increased HIF1α protein accumulation, whereas AKB stabilized HIF1α only at the higher dose (***inset***, Fig. 1e). Fgf23 mRNA expression was upregulated in response to FG-4592 and BAY85-3934, but was not significantly induced by AKB-6548 (Fig. 1f). In human U2OS cells, *TFRC* (Fig. S1a) and *FGF23* mRNA expression patterns (Fig. S1b) paralleled those of MPC2 cells. Collectively, these studies demonstrated that Fgf23 expression in osteocytes can be mediated by PHD inhibition.

To confirm the osteocyte response in vivo, we next utilized the conditional flox-*Fgf23* mouse model mated to the late osteoblast/osteocyte-specific dentin matrix protein-1 (Dmp1-cre).^31^ The flox-Fgf23/Dmp1-cre^+^ and cre^−^ mice were treated acutely as above with FG-4592. Compared to vehicle controls, treated mice in the cre^−^ group had significant elevations in plasma iFGF23 concentrations (Fig. 1g). The FG-4592-treated cre^+^ mice had an increase in iFGF23 that was ~50% lower than FG-4592-treated cre^−^ mice, and were similar to vehicle-treated cre^+^ mice, suggesting that a majority of the circulating iFGF23 in response to mimicked hypoxia is produced from osteocytes (Fig. 1g). Further, circulating serum EPO concentrations were similarly increased in both cre^−^ and cre^+^ mice treated with FG-4592, with no difference between genotypes (Fig. 1h). Importantly, these doses minimally impacted circulating iFGF23 in cre^+^ mice treated with FG-4592, suggesting that osteocytes were a primary and direct target of HIF-dependent FGF23 production.

### Osteocytes respond to HIF-PHI through genomic and transcriptional reprogramming

**Fig. 2:**
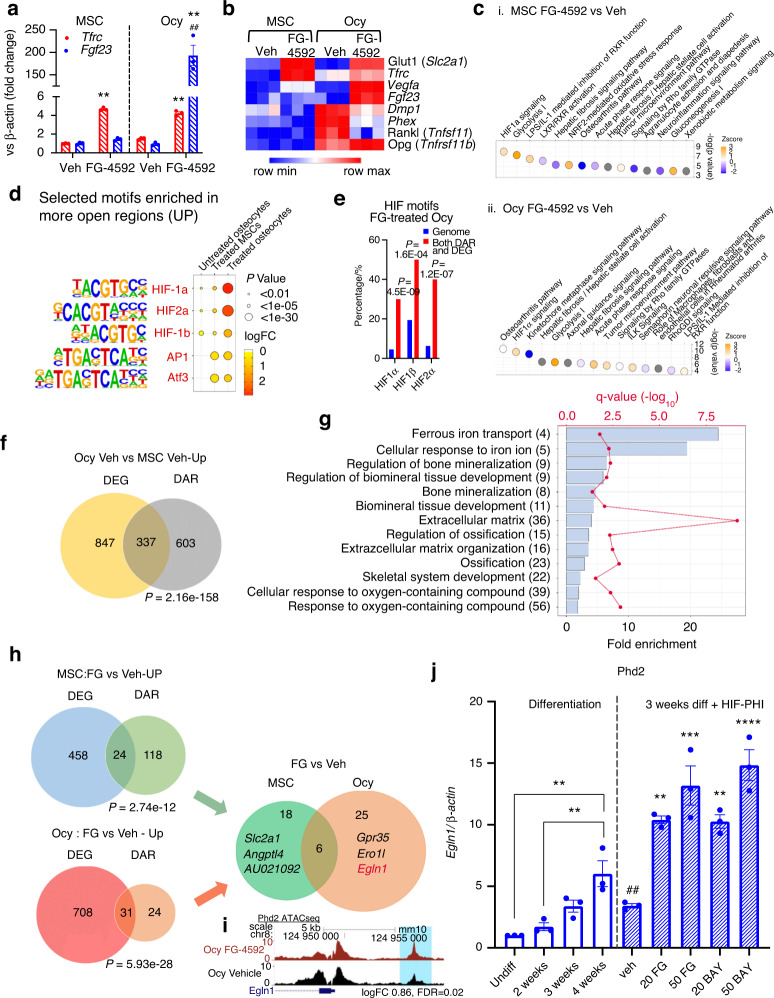
Osteocytes respond to HIF-PHI on the genomic level. **a** Transferrin receptor (*Tfrc*; red) and *Fgf23* (blue) mRNA expression was measured in mesenchymal stem cells (MSC; undifferentiated MPC2 cells) and osteocytes (Ocy; 3-week differentiated MPC2 cells) treated with vehicle or 50 μmol·L^−1^ FG-4592 for 48 hours (**P < 0.01 vs. veh; ^##^*P* < 0.01 vs. MSC, same treatment). **b** RNAseq heat map of specific HIF-responsive genes and selected genes involved in mineralization and bone turnover in untreated and treated MSC and Ocy. **c.i** Top 15 Canonical Pathways identified in RNAseq data by Ingenuity Pathway Analysis in FG-4592-treated MSC compared to vehicle-treated MSC and **c.ii** FG-4592-treated Ocy compared to vehicle-treated Ocy (orange Z-score indicates predicted pathway activation and blue indicates predicted pathway inhibition). **d** Selected motifs, including HIF motifs, enriched in more open regions of the genome in treated Ocy compared to treated MSC or untreated Ocy by ATACseq. **e** Integration of RNAseq and ATACseq showed significant enrichment of HIF motifs in more open regions of the genome associated with gene upregulation compared to the entire genome. **f** Venn diagram of differentially expressed genes (DEG) and differentially accessible regions (DAR) that overlap in Ocy vs. MSC, identifying 337 genes that have more chromatin within −10 kb of the TSS and have upregulated gene expression. **g** Gene Ontology (GO) Biological Processes enriched in Ocy compared to MSC (numbers in parenthesis are gene count). **h** Venn diagrams of differentially expressed genes (DEG) and differentially accessible regions (DAR) in FG-4592-treated MSC vs. vehicle-treated MSC and FG-4592-treated Ocy vs. vehicle-treated Ocy, identifying 24 and 31 genes, respectively, that have more chromatin within −10 kb of the TSS and have upregulated gene expression. Of these genes in each cell type, 6 overlapped, whereas 25 genes were unique to Ocy. Among the top 3 genes in Ocy was *Egln1* (encodes Phd2), a gene involved in oxygen sensing, and was thus examined further. **i** Representative ATACseq peaks of FG-4592-treated osteocytes (top track; crimson) compared to vehicle-treated osteocytes (bottom track; black). Highlighted in blue is the more open peak at −5 700 bp from the TSS of the Phd2 (*Egln1*) gene. **j** Phd2 expression across MPC2 cell osteogenic differentiation (**P < 0.01 vs. undifferentiated cells) and in 3-week differentiated MPC2 cells treated with FG-4592 or BAY85-3934 for 48 h (**P < 0.01, ***P < 0.001, ***P < 0.000 1 vs. veh; ^##^*P* < 0.01 vs. undifferentiated cells)

To test whether FGF23 was differentially responsive to changes in oxygen sensing during osteocyte maturation, qPCR analysis was used to examine the MPC2 cell line as undifferentiated MSCs or differentiated osteocytes (Ocy; at three weeks). With FG-4592 treatment, both the parent MSCs and differentiated osteocytes showed similar mRNA expressional changes in Transferrin receptor (*Tfrc* (red); Fig. 2a). In contrast, osteocytes increased *Fgf23* mRNA expression in response to FG-4592, whereas MSCs showed no change (blue; Fig. 2a). Next, to examine whether the gene expression changes were associated with HIF-PHI-mediated effects at a genomic level, parallel Assay for Transposase-Accessible Chromatin sequencing (ATACseq) and mRNA sequencing (RNAseq) was performed on undifferentiated MSCs and on 3-week differentiated osteocytes treated with FG-4592 (50 μmol·L^−1^) or vehicle, for 48 hours. Principle component analysis (PCA) of the ATACseq peaks showed distinct separation of MSCs and osteocytes, with some overlap between MSC treatment groups (Fig. S2a). RNAseq PCA showed distinct separation between MSCs and osteocytes, with no overlap between treatment groups (Fig. S2b). Both cell types showed rapid responses to mimicked hypoxia as indicated by detection of differentially accessible chromatin (Fig. S2c; Table S1, full list of each comparison in Tables S2–S4) and differentially regulated genes (Fig. S2d: Table S5, full list of each comparison in Table S6–S8). Candidate genes were next examined for expressional changes in MSCs and osteocytes (Fig. 2b). HIF responsive genes such as Glut1 (*Slc2a1*), Tfrc, and Vegfa showed similar elevations in MSCs and osteocytes with FG-4592 treatment (Fig. 2b), in parallel to the results above. Further, Fgf23 mRNA was upregulated, whereas genes involved in osteocyte mineral metabolism such as *Dmp1* and *Phex* showed significant downregulation. Genes known to be involved in bone turnover, such as *Opg* and *Rankl* mRNAs were down- and up-regulated, respectively. Importantly, these genes were only altered in response to hypoxia in osteocytes and not in undifferentiated cells (MSCs), suggesting a shift in transcriptional response to hypoxia with osteogenic differentiation.

We next performed Ingenuity Pathway Analysis (IPA) on the RNAseq transcripts from MSCs and osteocytes treated with FG-4592 compared to the vehicle-treated controls. The most significant canonical pathway identified by IPA in FG-4592-treated MSCs was the HIF1α Signaling pathway with a positive z-score (predicted pathway activation) (Fig. 2ci, full list in Table S9). The most induced pathway (ranked by *p*-value) identified in FG-4592-treated osteocytes was the Osteoarthritis Pathway, which had a positive z-score, consistent with the cells having a bone-related phenotype (Fig. 2cii, full list in Table S10). In accord with the ability of osteocytes to respond to changes in oxygen and iron handling, IPA of the RNAseq data showed HIF1α Signaling with a positive z-score (predicted pathway activation) as the second most enriched canonical pathway in FG-4592 treated osteocytes (Fig. 2cii). We next visualized the ATACseq accessibility peaks within and near the mouse *Fgf23* gene using the UCSC genome browser (Fig. S3). The presence of a −16 kb enhancer region known to contain HIF-responsive elements (HRE) was confirmed^32^ and several other peaks demarking open chromatin regions at the transcriptional start site and 3′ UTR were identified. Differential analysis did not detect significant changes in chromatin accessibility with differentiation or mimicked hypoxia (Fig. S3) however, suggesting that HIF activation of FGF23 transcription occurs in enhancer and promoter regions that are already accessible to these factors. HOMER transcription factor motif analysis of the ATACseq data identified three HIF motifs (HIF1α/β and HIF2α) modestly enriched in more accessible chromatin in osteocytes (‘untreated osteocytes’) compared to MSCs (Fig. 2d). In contrast, motif analysis of FG-4592 treated osteocytes showed that the known HIF transcription factor (TF) motifs were highly enriched in FG-4592-treated osteocytes compared to treated-MSCs or osteocytes alone. AP1 and Atf3 motifs were enriched in treated MSCs and osteocytes but were not present in untreated osteocytes. These data support that osteocytes demonstrate the ability to rapidly alter genomic accessibility, making these cells highly responsive/sensitive to mimicked hypoxia.

Integrative analysis of the ATACseq and RNAseq data showed that the three HIF motifs were significantly enriched in FG-4592-treated osteocytes in genes corresponding to both increased chromatin accessibility and upregulated gene expression in FG-4592-treated osteocytes compared to the background accessibility across the genome (Fig. 2e). This integrated data of differentially expressed genes (DEG) and differentially accessible regions (DAR) identified 337 upregulated genes associated with more open chromatin within −10 kb of the transcriptional start site (TSS) in osteocytes versus MSCs (Fig. 2f). Gene Ontology (GO) analysis of this data set showed enrichment for biological pathways known to mediate osteoblast differentiation and ossification processes, as well as processes related to cellular iron handling, and oxygen sensing/hypoxia (Fig. 2g). Taken together, these results demonstrate that osteocytes are highly sensitive to changes in pathways that respond to oxygen adaptation, which may, in part, be due to chromatin and gene expression changes that occur during differentiation from MSCs to osteocytes. Twenty-four upregulated genes in treated MSCs and 31 upregulated genes in treated osteocytes were associated with more open chromatin, and thus tested for overlapping and unique genes when comparing these data sets (Fig. 2h). We found that FG-4592 stimulated 25 distinct genes that were unique to osteocytes versus MSCs (Fig. 2h). Among these changes, the most significant loci for increased expression and accessibility unique to FG-4592-treated osteocytes included *Gpr35* (a G protein-coupled receptor known to be associated with HIF1^33^ and shown to regulate osteogenesis via the Wnt pathway^34^), *Ero1l* (an oxidoreductase involved in ER-stress induced apoptosis that is a known HIF-target gene^35^), and *Egln1* (encodes Phd2, a key hydroxylase regulating HIF turnover) (Fig. 2h and Table S11). Of note, Phd2 (*Egln1*) and Tfrc1 (Fig. S2e), as well as Glut1 and Vegfa mRNAs (from Fig. 2b) were increased in MSCs and osteocytes as determined by RNAseq, but Fgf23 was only elevated in osteocytes (Fig. 2b), confirming our in vitro studies above (see Fig. 2a). Additionally, a region 5 700 bp upstream of the *Egln1* TSS demonstrated increased accessibility in osteocytes in response to FG-4592 (Fig. 2i). Due to the facts that FGF23 is stimulated by hypoxia/anemia and that Phd2 is known to be upstream of HIF activity, this gene was further explored. Indeed, as MSCs temporally differentiate into osteocytes, Phd2 mRNA expression increased 7-fold (Fig. 2j; ‘Differentiation’). Ex vivo, osteocyte-enriched bone fragments from wild type mice were shown to have greater mRNA expression of Phd2 compared to Phd1 or Phd3 (Fig. S4a). Additionally, Phd2 mRNA expression was increased in osteocytes in response to the HIF-PHI FG-4592 and BAY 85-3934 (Fig. 2j; ‘3 weeks diff + HIF-PHI’), making Phd2 a candidate upstream regulator of FGF23 in response to changes in oxygen/iron utilization.

### The role of osteocyte Phd2 in vivo

**Fig. 3:**
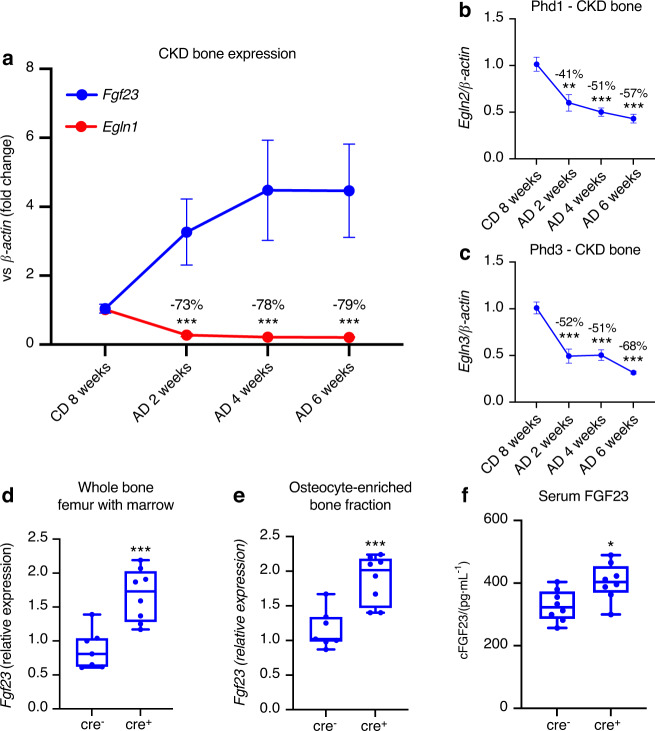
The role of osteocyte Phd2 in vivo. Wild type (WT) male mice were fed an adenine diet (AD) to induce CKD for 2, 4, or 6 weeks. Mice on casein diet for 8 weeks were used as controls. **a** Bone *Fgf23* (blue) and *Egln1*/Phd2 (red) mRNA expression was measured over diet duration. **b** Male Phd1 and **c** Phd3 expression in CKD bone. Numbers above timepoints indicate the % decrease in gene expression compared to the casein diet control (*n* = 3–4 mice/group; **P < 0.01; ***P < 0.001 vs. casein diet control). Fgf23 mRNA expression in **d** whole bone (including marrow), **e** osteocyte-enriched bone fractions, and **f** plasma total (*C*-terminal) FGF23 concentrations in mice with conditional deletion of Phd2 in osteocytes (Phd2-fl/fl/Dmp1-cre^+^) compared to mice without deletion (Phd2-fl/fl/Dmp1-cre^−^) (*n* = 6 mice/genotype; *P < 0.05, ***P < 0.001 vs. cre^−^ mice)

CKD causes a progressive disruption of bone mineral metabolism and we hypothesized that it may also lead to disordered oxygen sensing in bone. To test the role of Phds in CKD bone, wild-type male mice were placed on an adenine diet for 2, 4, or 6 weeks, or 8 weeks on a casein control diet. Plasma intact FGF23 concentrations were markedly elevated in adenine diet-fed mice compared to the casein diet control mice (Fig. S4b). Fgf23, Egln1 (Phd2), Egln2 (Phd1), and Egln3 (Phd3) mRNAs were measured in cortical bone (flushed of marrow) from femur and tibia. Fgf23 mRNA was significantly induced over time consistent with our previous results,^31^ whereas bone Egln1/Phd2 was downregulated in CKD mice (Fig. 3a). Compared to casein diet controls, Phd1 (Fig. 3b) and Phd3 mRNA expression (Fig. 3c) in CKD bone was also significantly reduced. These data demonstrate that CKD bone has dysregulation of oxygen-sensing mechanisms that may be contributing to elevated FGF23 levels seen in this disease. To test the role of Phd2 as a mediator bridging oxygen sensing and FGF23 expression in vivo, mice with conditional deletion of Phd2 in osteocytes (flox-Phd2/Dmp1-cre; as previously described^10^) were next tested. These mice are known to have increased osteocyte HIF1α protein due to loss of regulated turnover with deletion of Phd2, as well as high bone mass that is resistant to pathological challenge.^10^ In qPCR analyses, Fgf23 mRNA expression in whole bone (Fig. 3d) and osteocyte-enriched bone fractions (Fig. 3e) was significantly upregulated in the Dmp1-cre^+^ mice versus cre^−^ controls. Serum intact FGF23 concentrations were similar between cre^−^ and cre^+^ mice (Fig. S4c), whereas plasma total (*C*-terminal) FGF23 was found to be modestly elevated in the Dmp1-cre^+^ mice versus cre^−^ mice (Fig. 3f), supporting our hypothesis that Phd2 is upstream of FGF23 and is involved in FGF23 regulation in health and diseased bone in vivo.

### Testing Phd2 as a bridge for oxygen/iron sensing mechanisms to Fgf23 production

**Fig. 4:**
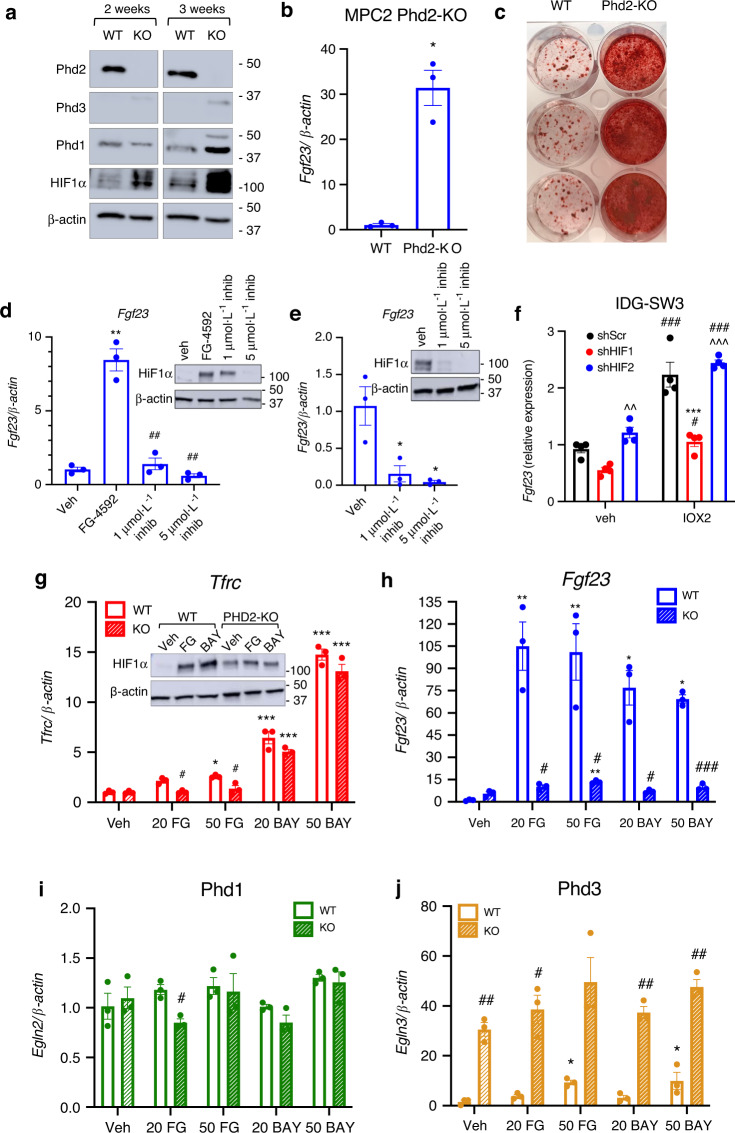
Phd2 bridges oxygen sensing mechanisms with Fgf23 production. A Phd2-KO cell line was generated in undifferentiated MPC2 cells using CRISPR/Cas9 technology and cells were differentiated for 2 or 3 weeks in osteogenic conditions. **a** Immunoblot of the Phd isoforms and HIF1α protein expression in 2- and 3-week differentiated WT and Phd2-KO cells. **b** *Fgf23* expression in 3-week differentiated WT and Phd2-KO MPC2 cells (*P < 0.05 vs. WT cells). **c** Alizarin red stain to visualize mineral deposition in 3-week differentiated WT and Phd2-KO MPC2 cells. **d** *Fgf23* gene expression and (**D**, inset) HIF1α protein expression in 3-week differentiated WT MPC2 cells treated with the HIF-PHI FG-4592 (Roxadustat) alone or with FG-4592 and 1 μmol·L^−1^ or 5 μmol·L^−1^ of the HIF1α inhibitor BAY 87-2243 for 48 h (**P < 0.01 versus vehicle; ^##^*P* < 0.01 versus FG treatment alone). **e** *Fgf23* expression and (inset) HIF1α protein expression in 3-week differentiated Phd2-KO cells treated with vehicle (DMSO) or with 1 μmol·L^−1^ or 5 μmol·L^−1^ of the HIF1α inhibitor BAY 87-2243 for 48 h (*P < 0.05 vs. veh). **f** *Fgf23* expression in IDG-SW3 cells transduced with shRNA against HIF1α or HIF2α in vehicle or IOX2-treated cells (***P < 0.001 vs. Scr control, same treatment, ^###^*P* < 0.001 vs. vehicle, same shRNA, ^^^*P* < 0.001 shHIF1 vs. shHIF2). **g** *Tfrc*, (**g**, inset) HIF1α protein, **h** *Fgf23*, **i** Phd1, and **j** Phd3 gene expression in 3-week differentiated WT (solid bars) and Phd2-KO (dashed bars) MPC2 cells treated for 24 h with 20 μmol·L^−1^ or 50 μmol·L^−1^ of the HIF-PHI FG (FG-4592; Roxadustat) or BAY (BAY85-3934; Molidustat) (*P < 0.05, **P < 0.01, ***P < 0.001 vs. veh, same genotype; ^#^*P* < 0.05, ^###^*P* < 0.001 WT vs. Phd2-KO, same treatment)

In light of the fact that Fgf23 mRNA expression was induced in vivo, and to rule out confounding compensatory effects from other iron maintenance systems, Phd2 was tested in the control of FGF23 in isolated systems in vitro. In this regard, two CRISPR/Cas9 approaches were used to reduce Egln1 in differentiated osteocytes. The first approach was a Phd2-knockout (KO) CRISPR targeting strategy where *Egln1*-guide RNA plasmids were transfected into undifferentiated MPC-2 cells to create ‘Phd2-KO’ MSCs. Initial screening of undifferentiated single-cell clones showed one clone (A2) had undetectable Egln1/Phd2 mRNA expression (Fig. S5a), which in parallel demonstrated elevated baseline Fgf23 mRNA concentrations (Fig. S5B). Taken together with our above data that FG-4592 does not stimulate Fgf23 mRNA in undifferentiated wild type MSCs, these findings support that Phd2 expression in MSCs likely suppresses Fgf23 transcription. Clone ‘A2’ was then isolated and differentiated into osteocytes using osteogenic media as previously described.^30^ Upon immunoblot, Phd2 protein was detectable in wild type (WT) cells but remained undetectable at 2 and 3 weeks of differentiation in the Phd2-KO cells, confirming CRISPR-mediated deletion (Fig. 4a). Wild type cells had low basal Phd3 protein expression, which modestly increased in Phd2-KO cells at 2 and at 3 weeks (Fig. 4a). Further, with loss of Phd2, basal Phd1 and HIF1α protein expression increased in Phd2-KO osteocytes at 2 and 3 weeks (Fig. 4a), potentially as compensatory responses to loss of Phd2-sensitive control of HIF turnover. The three-week differentiated Phd2-KO cells lost the ability to suppress basal Fgf23 transcription, as shown by a significant elevation of its mRNA with no treatment (Fig. 4b). Further, the 3-week differentiated Phd2-KO cells had markedly elevated secretion of intact (Fig. S5c) and total (*C*-terminal) FGF23 (Fig. S5d) compared to WT cells. Genetic deletion or chemical inhibition of *Egln1/*Phd2 in bone cells was previously associated with accelerated accumulation of bone mass/mineralization,^10^ and Phd2 knockdown in primary MSCs enhanced mineralization potential.^36^ As additional confirmation of complete ablation of *Egln1* from MPC2 cells, Phd2-KO cells grown in osteogenic media showed increased mineral content versus WT cells as visualized by Alizarin red staining (Fig. 4c).

To test whether increased Fgf23 expression in response to mimicked hypoxia was mediated through HIF1α, the HIF1α inhibitor BAY87-2243 was used on FG-4592-treated differentiated MPC2 osteocyte cells. FG-4592 treatment increased HIF1α protein and Fgf23 mRNA expression versus vehicle controls, and the cells treated additionally with increasing doses of BAY87-2243 had reduced HIF1α protein (*inset*, Fig. 4d) which was associated with dose-dependent and almost complete suppression of Fgf23 mRNA (Fig. 4d). Further, Phd2-KO cells showed baseline elevations in HIF1α protein in vehicle-treated cells (Fig. 4e, *inset)*. Additionally, direct HIF1α inhibition using BAY87-2243 reduced the accumulation of HIF1α in Phd2-KO cells as determined by immunoblot, which was associated with a dose-dependent suppression of Fgf23 mRNA expression (Fig. 4e and inset). To further support direct regulation of Fgf23 transcription by HIF1α, lentiviral delivery of shRNA against HIF1α or HIF2α was used in IDG-SW3 cells treated with vehicle or the HIF-PHI IOX2. These cells were previously characterized for HIF1α and HIF2α protein knockdown, which was shown to be effective.^10^ With IOX2 treatment, Fgf23 mRNA expression was upregulated (Fig. 4f), and cells with HIF1α knockdown had downregulated Fgf23 expression, whereas HIF2α knockdown had no effect the ability of IOX2 to induce Fgf23 expression, supporting that HIF1α was required for HIF-PHI-induced Fgf23 expression. Further, a CRISPR/Cas9 approach using a sgRNA lentiviral vector targeting Egln1 was transduced in IDG-SW3 cells. *Fgf23* mRNA expression was upregulated in the Phd2-KO viral targeted cells compared to control cells (Fig. S5e). Collectively, these data demonstrate that expression of Phd2 is required to suppress basal Fgf23 expression, and that this mechanism occurs via HIF1α-sensitive pathways.

To next test whether Phd2 was necessary for oxygen/iron mediated elevation of Fgf23, and to isolate the functional effects of Phd2 deletion in vitro, parent WT and Phd2-KO cells were differentiated for 3 weeks, then treated with FG-4592 or BAY85-3934 (20 or 50 μmol·L^−1^) for 24 hours. This analysis showed that Tfrc mRNA expression in FG-4592- and BAY85-3934-treated cells was modestly reduced with loss of Phd2, suggesting that overlapping Phd1 and −3-dependent pathways may be necessary to maintain iron utilization in osteocytes (Fig. 4g). As expected, FG-4592 and BAY85-3934 treatment of WT cells increased HIF1α expression compared to vehicle (*inset*, Fig. 4g). Consistent with results above, HIF1α expression was increased in vehicle-treated Phd2-KO cells, however treatment with FG-4592 or BAY85-3934 did not alter HIF1α accumulation (*inset*, Fig. 4g). In response to HIF-PHI treatment, Fgf23 mRNA expression was significantly attenuated (86%–91%) in all PHD2-KO cell groups compared to WT cells (Fig. 4h), further supporting that Phd2 is a critical mediator of Fgf23 production. Additionally, Phd1 mRNA expression was not significantly altered between WT and KO cells, and despite higher protein levels (Fig. 4a) there were no changes in Phd1 mRNA expression with HIF-PHI treatment (Fig. 4i). Phd3 mRNA expression was significantly increased in Phd2-KO cells (Fig. 4j), however as noted in the immunoblot blot of differentiated cells (Fig. 4a), this isoform is minimally detectable at the protein level in the basal state and its increase was not sufficient to reduce Fgf23. Thus, these studies demonstrate that Phd2 is required for suppression of Fgf23 in MSCs at baseline, and Phd2 deletion leads to HIF activation and increased FGF23 expression that was not compensated by Phd1 and Phd3 in mature osteocytes. Taken together, these studies identified Phd2 as a critical regulator of FGF23 expression within the osteocyte oxygen/iron sensing systems.

### Phd2 is required for iron-mediated suppression of Fgf23

**Fig. 5:**
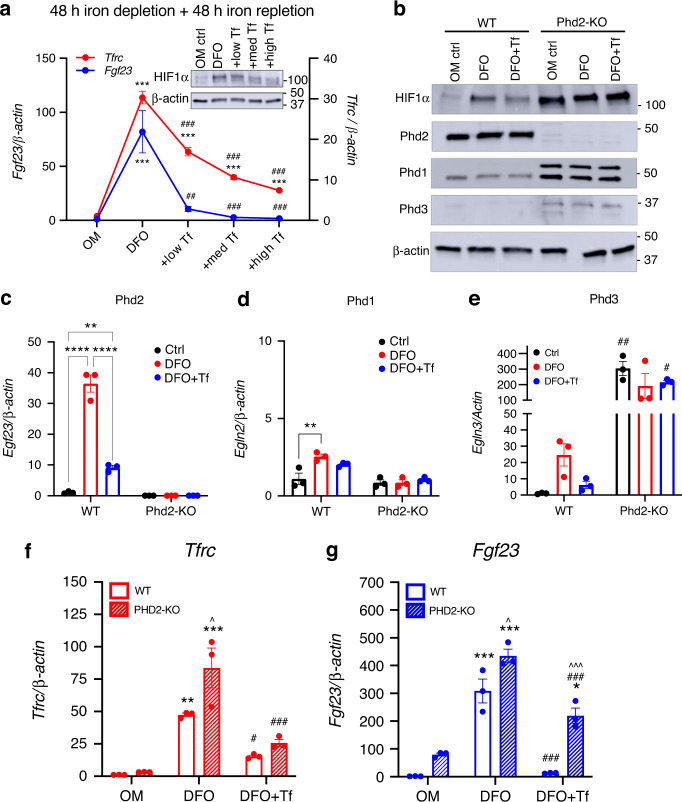
Phd2 is required for iron-mediated suppression of Fgf23. **a** Transferrin receptor (*Tfrc*; red) and *Fgf23* (blue) expression in WT 3-week differentiated MPC2 cells treated for 48 h with DFO, then repleted with iron using increasing doses of Holo-transferrin (Tf) in DFO-containing media for an additional 48 h (***P < 0.001 vs. OM ctrl; ^###^*P* < 0.001 vs. DFO alone). **a** inset HIF1α protein expression in MPC2 cells exposed to iron deficiency then repleted with iron using increasing doses of holo-Transferrin (Tf). **b** HIF1α, Phd2, Phd1, Phd3, and β-actin protein expression in 3-week differentiated WT and Phd2-KO cells exposed to iron deficiency for 48 h then iron repletion with high dose holo-Tf in DFO-containing media for an additional 48 h. **c** Phd2 (*Egln1*), **d** Phd1 (*Egln2*), and **e** Phd3 (*Egln3*) expression in 3-week differentiated WT and Phd2-KO cells exposed to iron deficiency for 48 h then iron repletion with high dose holo-Tf in DFO-containing media for an additional 48 h (**P < 0.01, ****P < 0.000 1; ^#^*P* < 0.05, ^##^*P* < 0.01 vs. genotype, same treatment). **f** *Tfrc* and **g** *Fgf23* expression in 3-week differentiated WT (solid bars) and Phd2-KO (dashed bars) cells exposed to iron deficiency for 48 h then iron repletion with high dose holo-Tf in DFO-containing media for an additional 48 h (**P < 0.01; ***P < 0.001 vs. OM ctrl, same genotype; ^#^*P* < 0.05, ^###^*P*< 0.001 vs. DFO, same genotype; ^*P* < 0.05,^^^*P* < 0.001 Phd2-KO vs. WT, same treatment)

During clinical anemia, FGF23 can be markedly elevated^37,38^ and it has been shown that FGF23 can be suppressed in ADHR patients receiving oral iron, effectively curing the ADHR renal phosphate wasting and metabolic bone disease.^19^ To test the concept that improved iron utilization directly controls circulating Fgf23, and that Phd2 is required for this process, an in vitro model of iron deficiency was employed. We previously demonstrated that acute iron depletion and repletion with holo-transferrin reciprocally regulates differentiated MPC2 osteocyte Fgf23 transcription.^26^ These analyses were expanded to WT and Phd2-KO cells using dose-dependent increases of holo-Transferrin during iron deficient conditions using the iron chelator deferoxamine (DFO). In this regard, cells that were maintained in DFO-containing media for the duration of the experiment (4 days) elicited increases in HIF1α protein (*inset*, Fig. 5a), Transferrin receptor (Tfrc), and Fgf23 mRNA compared to cells that remained in osteogenic media (OM) (Fig. 5a). After an additional 48 hours of iron repletion using dose-dependent increases in biologically available iron in the form of holo-Transferrin (holo-Tf) in DFO-containing media, cells with DFO + Tf had a dose-dependent downregulation of Tfrc and Fgf23 mRNAs (Fig. 5a) and reduced HIF1α protein accumulation (Fig. 5a, *inset*). Interestingly, even an “anemic” (+low Tf) concentration of holo-Tf highly suppressed Fgf23 expression ~87% and physiological levels of holo-Tf (+high Tf) suppressed Fgf23 by 98% and Tfrc expression by ~75% (Fig. 5a). These data demonstrate that osteocyte Fgf23 transcription is dose-dependently responsive to bioavailable iron.

Next, we tested the response to iron deficiency/repletion using the high dose of holo-Tf in WT and Phd2-KO cells. Immunoblot analysis showed cellular HIF1α protein accumulation during DFO-mediated iron deficiency, and a reduction of HIF in WT cells provided holo-Tf repletion (Fig. 5b). However, this effect was lost in Phd2-KO cells, regardless of iron status (Fig. 5b), and protein expression of the Phd1 and Phd3 isoforms was not altered with iron depletion/repletion (Fig. 5b). In response to DFO, of the Phd enzymes, Phd2 mRNA was the most significantly regulated by iron depletion and repletion (Fig. 5c) versus Phd1 and −3 (Fig. 5d, e). Finally, gene expression compensation from Phd1 (Fig. 5d) or Phd3 (Fig. 5e) was not observed in response to iron deficiency/repletion, which is also supported by immunoblot data of the same proteins (see Fig. 5b). Additionally, iron deficiency (DFO-treated cells) caused significant upregulation of Tfrc mRNA (Fig. 5f) and Fgf23 expression (Fig. 5g) in both WT and Phd2-KO cells. Iron repletion (DFO + Tf) reduced Tfrc expression to the same levels in both WT and Phd2-KO cells, however Fgf23 mRNA was only partially suppressed in the Phd2-KO cells compared to WT. These findings support that Phd2 is required for iron-mediated suppression of Fgf23 mRNA production (Fig. 5g). Thus, bioavailable iron directly regulates Fgf23 in osteocytes and the effects of iron repletion are lost when Egln1/Phd2 is deleted, revealing a critical role for this enzyme in bridging systemic changes in oxygen/iron sensing pathways to FGF23 production.

In sum, genomic, in vitro and in vivo findings demonstrate that Phd2 is a critical mediator of FGF23 production. Targeted deletion of this enzyme resulted in elevated Fgf23 in cells, and conditional deletion of Phd2 from osteocytes in vivo resulted in enhanced basal Fgf23 production. Further, unbiased studies showed that osteocytes rapidly respond to changes in oxygen/iron utilization through alterations in HIF-sensitive genomic accessibility that drive transcriptional reprogramming of adaptive pathways. Thus, osteocytes respond to changes in oxygen and iron via genomic and transcriptional mechanisms, and as a portion of this global response, osteocyte-expressed Phd2 is a required sensor to regulate Fgf23 production.

## Discussion

Osteocytes are essential for skeletal maintenance as they provide the necessary paracrine and endocrine factors for key structural and functional properties including bone density (Sost), resorption (RANK/RANKL), and mineralization (FGF23, DMP1, PHEX). Due to their unique localization in the skeleton between marrow and the periosteum, multiple systems for coordinating bone homeostasis are centralized within osteocytes. However, the critical upstream molecular mechanisms that bridge systemic changes in oxygen and iron utilization to FGF23 bioactivity have not been identified, as well as the full response of the osteocyte as a direct target cell for oxygen/iron utilization adaptation. Herein, we demonstrate that Phd2 is a novel link between oxygen sensing and FGF23 production in the osteocyte.

Studies by us and others support the relationships between HIF/oxygen sensing and FGF23, including chromosome immunoprecipitation (ChIP) analysis showing HIF binding to the *Fgf23* promoter and inducing Fgf23 expression,^17^ as well as the presence of Hypoxia Responsive Elements (HREs) in a putative *Fgf23* enhancer region.^32^ In our studies, treatment of wild type mice with HIF-PHI to mimic hypoxia, specifically FG-4592 (Roxadustat) and BAY85-3934 (Molidustat), increased Fgf23 mRNA expression and circulating intact FGF23 in vivo. Although the HIF-PHI do not specifically target a single PHD isoform, some have a greater affinity/preference for one PHD over others.^39^ Previous studies have highlighted this difference, as it was noted that BAY85-3934 is more potent in stabilizing HIF1α using an in vitro HIF1-hydroxylation assay than FG-4592, GSK1278863 (Daprodustat), and AKB-6548 (Vadadustat).^40^ Indeed, we showed the targeted effects of HIF-PHI on osteocytes in vivo using osteocyte conditional deletion of flox-*Fgf23* mice, which had suppressed circulating iFGF23 when treated with FG-4592, indicating that the osteocyte is the primary cell responsible for controlling Fgf23 expression during reduced oxygen and iron. Over longer time courses, EPO has been implicated in stimulating marrow FGF23 production.^41–43^ These findings were also consistent with our prior studies demonstrating that mice with late osteoblast/osteocyte deletion of flox-Fgf23 in the context of CKD also had lower circulating FGF23 concentrations.^31^ These mice also manifested elevated serum phosphate and hyperparathyroidism demonstrating key roles for the osteocyte in FGF23-mediated skeletal disease.

Osteocytes are deeply embedded in bone matrix, causing low oxygen tension in the cellular milieu. Several studies have shown hypoxia preconditioning of MSCs improves proliferation, self-renewal capacity, and osteogenic differentiation potential,^44,45^ demonstrating hypoxia as a key factor for adequate stem cell function. Through unbiased genome-wide RNAseq and ATACseq in combination with FG-4592 treatment, we identified multiple, directly modifiable pathways associated with oxygen and iron sensing in osteocytes. In this regard, HIF1α Signaling and the Osteoarthritis Pathway were highly and rapidly induced, demonstrating that hypoxia is a critical factor for osteocyte function. Further, we demonstrated that as MSCs differentiate into osteocytes, HIF sites become more accessible with mimicked hypoxia, indicating that as the osteoblast lineage matures, osteocytes are poised to adapt to changes in oxygen and iron. This concept is supported by previous studies demonstrating that hypoxia enhances osteogenesis^46,47^ and promotes the transformation of osteoblasts towards osteocytes.^9^ Our genome-wide approach using integrated chromatin accessibility and gene expression identified gene regulatory changes in response to a hypoxia mimetic, including both genomic and transcriptional changes of *Egln1* (Phd2) in osteocytes, that was also associated with parallel increased Fgf23 mRNA. Using CRISPR to target Phd2, our data support that Phd2 may play a role in suppressing Fgf23 in MSCs, as deletion of this enzyme resulted in basally elevated Fgf23 mRNA. However, MSCs had less open chromatin accessibility at HIF sites during mimicked hypoxia compared to osteocytes and did not express Fgf23 during FG-4592 treatment. Therefore, changes in oxygen/iron sensing that occur during MSC to osteocyte maturation are likely critical for systemic control of overall skeletal function

Several studies have further developed an understanding of the role of PHDs in a variety of bone cells, which may be dependent upon bone cell developmental stage. In this regard, a comprehensive analysis of the role of the Phd1-3 isoforms in osteoblast lineage cells using the progenitor cell targeted Osx-cre was performed by Wu, et al., showing that no single deletion of a Phd isoform in osteoblast progenitors had effects on bone mass. However, using in vivo conditional deletion of all three isoforms conjointly, the resulting mice showed increased bone mass,^48^ which was in contrast to results generated in osteoblast lineage flox-*Phd2*/Col1a2-cre deleted mice which showed reduced bone mass.^49^ In this regard, Phd2 may be critical for bone maintenance as genetic knockdown of Phd2 or inhibition of Phds using the HIF-PHI IOX2 was shown to enhance bone formation in periosteum-derived cell implants in vivo.^50^ More recently, Phd2 deletion in osteocytes produced mice with increased bone mass (BMD) and strength at basal levels in vivo which was associated with lower Wnt-inhibitor Sclerostin (Sost) expression and resistance to skeletal unloading and ovariectomy associated with low bone mass phenotypes.^10^ Thus, in combination with the present work, osteocytes may coordinate bone formation with biomineralization through control of FGF23 in parallel with genes that build bone.

We determined that whole bone contained the highest mRNA expression of Phd2, versus Phd1 and −3. Further, osteocytes also have been reported to express the highest Phd2 levels compared to related cell types, such as osteoblasts, reflecting the possibility that this isoform serves as a key mediator of HIF activation within their hypoxic milieu.^10^ To date, the osteocyte mediators responsive to changes in oxygen and iron sensing upstream of HIF control of FGF23 have not been tested. Mice with osteocyte-specific deletion of Phd2 had modestly increased transcription and secretion of *C*-terminal FGF23, however there were no differences in intact FGF23 versus the mice acutely injected with HIF-PHI that had higher FGF23 mRNA and plasma protein. This could potentially be due to long-term compensatory responses for both oxygen/iron handing and phosphate homeostasis that suppress FGF23 over the lifespan as the Phd2 conditional deletion in the mice occurred since birth. Future studies could use an inducible Dmp1-cre to study acute effects and avoid any long-term confounding factors. A recent study of patients with HIF2A gain-of-function mutations^51^ had some parallel phenotypes as our Phd2-KO cells and Phd2 conditional mice that have elevated osteocyte HIF1α (no change in HIF2α expression^10^) and increased *C*-terminal FGF23. These patients had elevated circulating EPO, and we^41^ and others^43^ have shown that that elevated EPO concentrations can stimulate FGF23 in bone and bone marrow, thus the elevated *C*-terminal FGF23 may be via elevated EPO in these patients. Our study is able to distinguish direct effects in bone/osteocytes, where mice with conditional deletion of Phd2/HIF1α accumulation in osteocytes specifically, had elevated *C*-terminal FGF23 with no change in erythropoiesis.^10^ In addition, using HIF shRNA, we determined in vitro in differentiated IDG-SW3 cells that during Phd2 inhibition, targeted shRNA knockdown of HIF1α inhibited FGF23 mRNA production whereas HIF2α shRNA did not affect FGF23 transcription. Although we cannot completely rule out HIF2α activity in the production of FGF23 in the absence of HIF1α, our data point to HIF1α being predominantly involved in osteocytes.

Herein, we showed ablation of Phd2 by CRISPR in MSCs induces basal expression of Fgf23 mRNA in vitro, and that differentiated Phd2-KO osteocytes have increased Fgf23 that is completely suppressed by HIF1α inhibition. This finding supports that osteocytic Egln1/PHD2 deletion may function as the key mediator of Fgf23 expression during anemia, which results in hypoxia and elevated HIF. Our studies did not consider effects of elevated FGF23 on mineralization potential. Future studies could inhibit FGF23 in the presence or absence of elevated HIF1α to test for a direct role for FGF23 in the biomineralization of these cells. Although we showed that CRISPR deletion of Phd2 had marked effects on Fgf23 expressional control in response to mimicked hypoxia and anemia, we did not directly investigate the consequences of Phd1 or Phd3 deletion in vitro. Our data demonstrate that Phd1/3 protein expression are increased following CRISPR deletion of Phd2, but FGF23 is still increased basally, thus if to any extent, these enzymes likely play only minor roles in FGF23 regulation. In addition to changes in FGF23, the Phd2-KO cells also had enhanced mineralization when differentiated into osteocytes, which mimics the increased in vivo bone mass phenotype in the conditional osteocyte-deleted flox-Phd2 mice as previously described.^10^ Although we did not determine whether this is due to increased amount of organic matrix available or if the available matrix is more densely mineralized, our current results recapitulate previous findings in vitro using primary cultures of MSCs,^36^ and show that bone mass and biomineralization may be controlled symmetrically by osteocyte Phd2-mediated events.

The molecular mechanisms guiding Fgf23 production in the osteocyte are complex, with among others, hormonal, mineral ion, steroid hormone, and inflammation influencing transcription and translation. In this regard, we previously demonstrated that anemia in WT mice and a mouse line carrying an ADHR FGF23 stabilizing mutation responded to iron deficiency anemia (IDA) by increasing Fgf23 mRNA.^3^ Anemia is also very common in CKD, where 75% of patients by disease stage 4-5 have anemia and concomitant marked elevations in FGF23. Indeed, we previously demonstrated that correcting anemia in a CKD model using recombinant EPO, FG-4592, and BAY85-3934 reduced FGF23.^25,26^ In clinical studies in ADHR patients, iron therapy suppressed FGF23 expression and improved clinical disease manifestations.^19^ However, whether this was due to direct actions of FGF23 from the osteocyte was unclear. It was recently shown clinically that FGF23 mediated the risks of heart failure and mortality associated with iron deficiency in CKD patients.^29^ Herein, we used an in vitro model of osteocyte iron deficiency with iron repletion via holo-transferrin demonstrated direct actions on the osteocyte to regulate Fgf23 expression, and further, that Phd2 is necessary for iron-mediated suppression of Fgf23. Additionally, a clinical trial using ferric citrate, an iron-containing phosphate binder, to treat IDA in CKD patients showed circulating FGF23 levels were reduced,^52^ highlighting the role of iron provision to suppress FGF23, potentially through direct actions on osteocytes. Therapies involving intravenous iron delivery reduced circulating FGF23 in anemic patients^38,52,53^ but may manifest side effects, including oxidative stress, liver iron overload, and cardiovascular disease.^54^ Thus the present work suggests that directly modulating PHD2 function in osteocytes may reduce FGF23 to prevent severe manifestations of FGF23 over expression, but not to a level causing hyperphosphatemia which is associated with negative patient outcomes.^55,56^

In conclusion, these studies demonstrate that Phd2 is a critical sensor in osteocytes that links the extracellular milieu with FGF23. These findings may be translatable for diseases that involve both oxygen deprivation/anemia and phosphate handling such as CKD by providing novel skeletal targets that influence bone structure and function in disease.

## Materials and Methods

### Animal studies

Animal studies were approved by, and performed, according to the Institutional Animal Care and Use Committee (IACUC) of the Indiana University School of Medicine, and complied with the NIH guidelines for the use of animals in research. C57BL/6 mice were purchased (Jackson Labs) and acclimated prior to study. Dmp1-cre/flox-Fgf23 mice were bred in house.^57^ Osteocyte-specific deletion of PHD2 was obtained by crossing *Phd2*^*fl/fl*^ mice^58^ with Dmp1-cre transgenic mice^59^ (*Dmp1-Cre*^*+*^ *Phd2*^*fl/fl*^). *Dmp1-Cre*^*−*^ *Phd2*^*fl/fl*^ littermates were used as control in all experiments; analysis was performed on 8-week-old male mice unless stated otherwise. *Dmp1-Cre*^*−*^ *Phd2*^*fl/fl*^ mice (100% C57BL/6 background) were bred in conventional conditions, and housing and experimental procedures were approved by the Institutional Animal Care and Research Advisory Committee of the KU Leuven (ethical approval number P214/2013). Mice were euthanized by CO_2_ inhalation/cervical dislocation, and blood was collected by cardiac puncture for serum and plasma (collected in EDTA tubes). Baseline bleeds were taken 2–3 days prior to the first HIF-PHI injection or diet administration, collecting less than 5% of the total blood volume to mitigate potential effects on the parameters tested.^60^

### Rodent diets

Mice were fed normal rodent chow prior to experimental diet administration. The adenine diet model was used to induce renal failure in male wild type mice according to previous protocols.^31^ Significant sex effects have been shown with the provision of adenine-containing diets, with males developing disease phenotypes more rapidly.^61^ At 8 weeks of age, mice were transferred to a diet containing 0.2% adenine (TD.160020; Envigo) and euthanized after 2, 4, or 6 weeks on diet. Male mice on a casein diet (0.9% phosphate and 0.6% calcium, TD.150303 Envigo) for 8 weeks were used as the control. Diets and water were provided ad libitum.

### In vivo HIF-PHI treatment

8-week-old wild type female mice received intraperitoneal (i.p.) injections of either vehicle (5%DMSO/45%PEG300/50%ddH2O) or 50 mg·kg^−1^ of FG-4592 (‘FG’, Roxadustat; SelleckChem) or BAY 85-3934 (‘BAY’, Molidustat; SelleckChem) every other day for 5 days (3 injections total). Female flox-Fgf23/Dmp1-cre^+^ and cre^−^ mice (10-11 weeks old) were injected with 70 mg·kg^−1^ of FG-4592 every other day for 5 days (3 injections total). In all studies, tissues were harvested 4 hours after the final injection.

### Serum biochemical parameters

Plasma/serum FGF23 was assessed using rodent-specific commercial ELISAs for bioactive, intact FGF23 (iFGF23; Quidel, Inc.) or ‘*C*-terminal’/total FGF23 (‘cFGF23’; Quidel, Inc). Plasma EPO was measured using the Mouse Epo Quantikine ELISA (R&D Systems). Serum intact FGF23 in Dmp1-cre/Phd2-fl/fl mice was measured using the Kainos FGF23 ELISA kit (Kainos Laboratories, Inc).

### Isolation of osteocyte-enriched bone fractions

i. Wild type C57BL6 mice: Femurs and tibiae were dissected from 10-week-old mice. Epiphyses were cut off and bone marrow was flushed out of bones. Bones were then minced into several pieces and underwent two collagenase digestions (2 mg·mL^−1^ in αMEM) for 20 minutes followed by one 5 mM EDTA digestion for 20 minutes (all at 37 °C) with three HBSS washes in between each step. Bone chips were placed on collagen coated plates using rat Type I Collagen (Fisher Scientific) in αMEM supplemented with 25 mmol·L^−1^ penicillin-streptomycin (Sigma-Aldrich, St. Louis, MO, USA), 2.5% fetal bovine serum (FBS, Hyclone) and 2.5% bovine calf serum (BCS, Hyclone). Bone chips sat undisturbed for 7 days, then half of the media was changed, and cells were incubated for an additional 48 h before RNA analyses.
ii. Dmp1-cre/flox-Phd2 mice: Tibiae and femurs of 7–9-week-old mice were cleaned to remove muscle and connective tissue. Subsequently, bones were incubated in 2 mg·mL^−1^ collagenase (in αMEM with 2 mmol·L^−1^ glutaMAXTM-1, containing 100 U·mL^−1^ penicillin and 50 μg·mL^−1^ streptomycin; all from Gibco) for 30 min at 37 °C. Epiphyses were cut, bone marrow was flushed and bone was cut into smaller pieces. These fragments were incubated in 1 mg·mL^−1^ collagenase mixture for 40 min at 37 °C and this cell suspension was discarded. The remaining bone chips were washed with PBS and incubated for 40 min at 37 °C with 5 mmol·L^−1^ EDTA in PBS. Cell suspension was again discarded and the bone fragments were finally incubated with 1 mg·mL^−1^ collagenase mixture for 50 min at 37 °C. Cells were collected, passed through a 70 μm nylon mesh and washed twice. This osteocyte-enriched bone fraction was directly lysed for RNA analysis.

### Cell culture

Human U2OS cells (ATCC) were plated at a density of 1.5 × 10^5^ cells per well in 6- or 12-wll plates and incubated overnight before treatment. Murine progenitor cells clone 2 (MPC2),^30^ a conditionally immortalized mesenchymal stem cell line, were acquired from William Thompson (IUSM) and cultured in αMEM (Invitrogen, Thermo-Fisher Scientific) supplemented with 10% fetal bovine serum (FBS; Hyclone), 25 mmol·L^−1^ L-glutamine, and 25 mmol·L^−1^ penicillin-streptomycin (Sigma-Aldrich, St. Louis, MO, USA) at 33 °C and 5% CO2 to proliferate. Cells were plated at a density of 1.0 × 10^5^ cells per well in 6- or 12-well plates and incubated overnight before being transferred to a 37 °C incubator for osteogenic differentiation and cultured in maintenance media supplemented with mineralization conditions of 4 mmol·L^−1^ beta-glycerophosphate and 50 μg·mL^−1^ ascorbic acid. Cells were differentiated for 2 or 3 weeks where indicated with osteogenic media changes every 2-3 days. IDG-SW3 cells, kindly provided by L. Bonewald,^62^ were expanded at permissive conditions (33 °C in nucleoside-supplemented αMEM with 10% FBS, 100 U·mL^−1^ penicillin, 50 μg·mL^−1^ streptomycin and 50 U·mL^−1^ IFN-γ) on rat-tail collagen type I-coated plates (0.15 mg·mL^−1^ collagen in 0.02 mol·L^−1^ acetic acid). IFN-γ was from Thermo Fisher Scientific, rat-tail collagen type I from BD Biosciences and other reagents were from Gibco. For osteogenic differentiation, IDG-SW3 cells were expanded and upon confluency, medium was switched to osteogenic differentiation medium, consisting of growth medium supplemented with 50 μg·mL^−1^ ascorbic acid and 4 mmol·L^−1^ β-glycerophosphate (both Sigma-Aldrich) and cultured at 37 °C.

### In vitro studies

i. *HIF-PHI treatment:* U2OS cells and 2-week differentiated MPC2 cells were treated for 4 h (for HIF1α protein), 24 h or 48 h (for RNA) with vehicle (DMSO), 20 μmol·L^−1^ or 50 μmol·L^−1^ of the HIF-PHIs FG-4592 (Roxadustat; SelleckChem), BAY 85-3934 (Molidustat; SelleckChem), or AKB-6548 (AKB, Vadadustat; MedKoo) as previously reported.^63^ PHD2-KO cells and corresponding WT cells were differentiated for 3 weeks and treated with 20 μmol·L^−1^ or 50 μmol·L^−1^ FG-4592 or BAY85-3934 for 24 h. Cells were then washed with PBS and harvested for RNA and protein analysis.
ii. *HIF inhibitor studies:* 3-week differentiated WT MPC2 cells were pre-treated with 50 μmol·L^−1^ of FG-4592 for 1 h to increase HIF1α, then treated with 1 μmol·L^−1^ or 5 μmol·L^−1^ of the HIF inhibitor BAY 87-2243 (SelleckChem) for 48 h. 3-week differentiated MPC2 PHD2-KO cells were treated with 1 μmol·L^−1^ or 5 μmol·L^−1^ of BAY 87-2243 for 48 h. Cells were then washed with PBS and harvested for RNA and protein analysis.
iii. *Model of iron deficiency:* WT and PHD2-KO MPC2 cells differentiated for 3 weeks were incubated in osteogenic media (OM) alone, or OM containing 50 μmol·L^−1^ deferoxamine (DFO) for 48 hours, after which one set of cells was incubated with DFO media containing increasing levels of biologically available iron holo-Transferrin (0.2, 0.6, 1 mg·mL^−1^ as low, med and high holo-Tf, respectively), making up for total protein amount with apo-Transferrin, 1 mg·mL^−1^ holo-Tf and 0.2 mg·mL^−1^ apo-Tf, respectively,^64^ while another set of cells remained in ‘iron deficient’ DFO-containing media for an additional 48 hours. Cells were then washed with PBS and harvested for RNA or protein analysis.
iv. *CRISPR/Cas9 genome editing in MPC2 cells:* Phd2-KO plasmid was purchased from Santa Cruz (sc-431081) which contained 3 different plasmids combined into 1 vial, each with its own unique gRNA targeting exon 1, 2, or 3 of the murine *Egln1*/Phd2 gene as well as expression of Cas9 and GFP. The guide sequences for Phd2 were as follows: 5′- GTACTTCATGAGGGTTACGC −3′, 5′- CGGCAGTACTGCGAGCTGTG −3′, 5′- ACATAGCCTGTTCCGTTGCC −3′. Using the Fugene protocol and reagent, 8.8 μg of plasmid were transfected into a flask of proliferating MPC2 cells at 33 °C. Forty-eight hours post-transfection, the cells were washed with PBS, trypsinized, resuspended in media, and filtered through a 70 μm cell strainer. GFP-positive cells were single-cell sorted by FACS into 96-well plates (1 cell per well) containing MPC2 maintenance media supplemented with 20% FBS and filtered conditioned media from mostly confluent flasks of MPC2 cells. Single clones were expanded and screened for Phd2 expression by qRT-PCR and protein immunoblotting.
v. *HIF shRNA knockdown in IDG-SW3 cells*: After 7 days of osteogenic induction, when Dmp1 gene expression was upregulated, cells were treated with either DMSO (1/5 000 dilution) or the HIF-PHI IOX2 (10 μmol·L^−1^) to inhibit PHDs. To silence HIF-1α or HIF-2α in IDG-SW3 cells, we transduced these cells with a lentivirus carrying a shRNA against HIF-1α or HIF-2α (shHIF-1α and shHIF-2α, kindly provided by P. Carmeliet; MOI 10) in the presence of 8 μg·mL^−1^ polybrene (Sigma-Aldrich). A lentivirus carrying a nonsense scrambled shRNA sequence (shScr) was used as a negative control (MOI 10). After 24 h, virus-containing medium was changed to normal culture medium and 48 h later, cells and conditioned medium were used for further experiments.
vi. *CRISPR/Cas9 genome editing in IDG-SW3 cells*: To silence Phd2 in IDG-SW3 cells using CRISPR-Cas9, we transduced these cells with a lentivirus carrying a plasmid containing the Cas9 enzyme (lentiCRISPR v2; Addgene) and a sgRNA against Phd2 (5′-GCCTGGGTAACAAGCAACCA-3′). A nonsense scrambled sgRNA (5′-GCTGATCTATCGCGGTCGTC-3′) was used as a negative control. After 24 h, virus-containing medium was changed to normal culture medium and cells were selected with puromycin (0.3 μg·mL^−1^) for 7 days before they were used in subsequent experiments.
vii. *FGF23 protein secretion in Phd2-KO cells*: undifferentiated WT and Phd2-KO cells were seeded at 1×10^5^ cells per well into 6-well plates and grown to 90% confluency. Cells were then differentiated in osteogenic media for 21 days. Media was aspirated and cells were washed with 1X PBS, then osteogenic media was replaced and allowed to incubate 48 hours. Media was collected and centrifuged to remove unattached cells and debris. Media was concentrated in Amicon Ultra Centrifugal Filters (Milipore) and stored at −80 °C. Adherent cells were washed twice with 1X PBS then lysed with 300 µL of 1X Lysis buffer (Cell Signaling Technologies, Inc., Danvers, MA, USA) with 4-(2-aminoethyl) benzenesulfonyl fluoride hydrochloride (AEBSF) protease inhibitor (1 μg·mL^−1^) according to the manufacturer’s directions (Sigma-Aldrich, Inc.). Total cell lysate protein concentrations were determined with the Better Bradford Kit (Thermo-Fisher Scientific) according to the manufacturer’s instructions. Secreted FGF23 protein was assessed using both the rodent-specific ‘intact’ FGF23 (‘iFGF23’) and ‘*C*-terminal’ (or ‘total’) ‘cFGF23’ ELISAs (Quidel Laboratories, Inc.) and normalized to total protein concentration.

### mRNA sequencing (RNAseq)

MPC2 cells were differentiated for 3 weeks in osteogenic media or plated in an undifferentiated state at 33 °C (see cell culture methods above), then treated for 48 hours with 50 μmol·L^−1^ of the HIF stabilizer FG-4592 (SelleckChem) or vehicle (DMSO). Total RNA was extracted (with a DNase step) using the same methods as described in “RNA preparation” section of methods. Total RNA was evaluated for its quantity and quality using Agilent Bioanalyzer 2100. For RNA quality, a RIN number of 7 or higher is desired. The RIN number for all of the samples was 10. 100 ng of total RNA was used. cDNA library preparation includes mRNA purification/enrichment, RNA fragmentation, cDNA synthesis, ligation of index adaptors, and amplification, following the KAPA mRNA Hyper Prep Kit Technical Data Sheet, KR1352–v4.17 (Roche Corporate). Each resulting indexed library was quantified and its quality accessed by Qubit and Agilent Bioanalyzer, and multiple libraries pooled in equal molarity. The pooled libraries were denatured and neutralized before loading to NovaSeq 6000 sequencer at 300 pmol·L^−1^ final concentration for 100b paired-end sequencing (Illumina, Inc.). Approximately 30–40 M reads per library were generated. A Phred quality score (Q score) was used to measure the quality of sequencing. More than 90% of the sequencing reads reached Q30 (99.9% base call accuracy). The sequencing data were first assessed using FastQC (Babraham Bioinformatics, Cambridge, UK) for quality control. The reads were mapped to the mouse genome mm10 using STAR (v2.7.2a).^65^ RNAseq aligner with the following parameter: “--outSAMmapqUnique 60”. Uniquely mapped sequencing reads were assigned to Gencode M22 gene using featureCounts (v1.6.2)^66^ with the following parameters: “–p –Q 10 -O”. The data was filtered using read count >10 in at least 3 of the samples, normalized using TMM (trimmed mean of M values) method and subjected to differential expression analysis using edgeR (v3.20.8).^67^ Differentially expressed genes (DEGs) were identified given the cutoffs, |log_2_FC | > 1 and false discovery rate (FDR) < 0.05. Gene Ontology and KEGG pathway functional analysis was performed on up-regulated and down-regulated DEGs, respectively, using DAVID.^68^ Canonical pathways enriched in DEGs were determined through Ingenuity Pathway Analysis (IPA) (QIAGEN Inc., https://www.qiagenbioinformatics.com/products/ingenuity-pathway-analysis).^69^ *n* = 3 samples per condition.

### Assay for Transposase-Accessible Chromatin sequencing (ATACseq)

Cells used for ATAC sequencing were plated, differentiated, and treated identical to samples for RNAseq (see above). Cells were washed twice in 1X PBS, then dissociated with trypsin (Hyclone) for 5 minutes. Cells were resuspended in ice cold 1X PBS, dead cells were removed and processed for ATAC. Assay for transposase-accessible chromatin with high-throughput sequencing was performed according to the published protocol.^70^ Briefly, cells were collected in cold PBS and cell membranes were disrupted in cold lysis buffer (10 mmol·L^−1^ Tris–HCl, pH 7.4, 10 mmol·L^−1^ NaCl, 3 mmol·L^−1^ MgCl2 and 0.1% IGEPAL CA-630). The nuclei were pelleted and resuspended in Tn5 enzyme and transposase buffer (Illumina Nextera® DNA library preparation kit, FC-121-1030). The Nextera libraries were amplified using the Nextera® PCR master mix and KAPA biosystems HiFi hotstart readymix successively. AMPure XP beads (Beckman Coulter) were used to purify the transposed DNA and the amplified PCR products. All libraries were sequenced on a 100 cycle paired-end run on an Illumina NOVAseq instrument. The resulting ATACseq libraries were sequenced on Illumina NovaSeq 6000 at CMG of Indiana University School of Medicine and paired-end 50 bp reads were generated. Illumina adapter sequences and low-quality base calls were trimmed off the paired-end reads with Trim Galore v0.4.3. Bowtie2^71^ was used for ATACseq reads alignment on the mouse genome (mm10). Duplicated reads were removed using Picard [Broad Institute. (Accessed: 2018/02/21; version 2.17.8). “Picard Tools.” Broad Institute, GitHub repository. http://www.broadinst]. Low mapping quality reads and mitochondrial reads were discarded in further analysis. Peak calling of mapped ATACseq reads were performed by MACS2^72^ with a Bonferroni adjusted cutoff of p-value less than 0.01. Peaks called from multiple samples were merged. Merged peaks overlapping with ENCODE blacklist regions^73,74^ were removed to form a final set of regions. Reads overlapping with these regions in different samples were counted by pyDNase.^75^ The data was filtered using at least 10 cut counts in more than one of the samples, normalized using TMM (trimmed mean of M values) method and subjected to differential analysis using edgeR (v3.24.3).^67,76^ One sample with quality issues was removed from analysis. The differentially accessible regions (DARs) were determined by FDR < 0.05. Enrichment analysis on sequence motifs within DARs was performed using Homer based on FDR < 0.05.^77^ For integration of RNA-seq and ATAC-seq, we connected an open chromatin with one gene if the open chromatin locates within 10 kb upstream of the gene.

### Immunoblotting

Cells were lysed with 300 µL 1X Lysis buffer (Cell Signaling Technologies, Inc., Danvers, MA, USA) with 1 μg·mL^−1^ 4-(2-aminoethyl) benzenesulfonyl fluoride hydrochloride (AEBSF) protease inhibitor (Sigma-Aldrich, Inc.). Total cell lysate protein concentrations were determined with the Better Bradford Kit (Thermo-Fisher Scientific) according to the manufacturer’s instructions. Western blot analysis was performed with 50 µg of MPC2 or 40 µg of U2OS cellular lysates. The blots were incubated with primary antibody to hypoxia-inducible factor 1-alpha (HIF1α) (NB100–449; Novus Biologicals, Littleton, CO, USA) Phd1 (Abcam; ab108980), Phd2 (Novus; NB100-2219), Phd3 (Novus: NB100-303) overnight, then incubated with secondary antibody at 1:2 000 (anti-rabbit–horseradish peroxidase [HRP]; Cell Signaling Technologies). Blots were stripped using SDS-glycine and reprobed with 1:15 000 anti-β-actin-HRP (A3854; Sigma-Aldrich). Detection was performed using the ECL Prime Western Blotting Detection Reagents (Amersham-GE Healthcare, Pittsburgh, PA, USA) and the GE AB1600 digital imager.

### RNA preparation and quantitative RT-PCR (qPCR)

Cortical bone from femur and tibiae (marrow flushed) and digested bone chips were harvested and homogenized in 1 mL of Trizol reagent (Invitrogen/ThermoFisher Scientific) according to the manufacturer’s protocol, then further purified using the RNeasy Kit (Qiagen, Inc). MPC-2 and U2OS cells were lysed in RLY buffer (Bioline, Inc.). Total RNA from lysates was prepared using the Isolate II RNA Mini Kit (Bioline, Inc.). RNA samples were tested with intron-spanning primers/probe specific for mouse or human Fibroblast growth factor-23 (*Fgf23*) and Transferrin receptor (*Tfrc*) and mouse Phd1 (*Egln2*), Phd2 (*Egln1*), Phd3 (*Egln3*) mRNAs; Mouse or human *Gapdh* or *β-actin* was used as an internal control for RT-qPCR (Applied Biosystems/ThermoFisher Scientific). The TaqMan One-Step RT-PCR kit was used to perform the qPCR reactions under cycling conditions: 30 min 48 °C, 10 min 95 °C, followed by 40 cycles of 15 s 95 °C and 1 min 60 °C. The data were collected using a StepOne Plus system (Applied Biosystems/ThermoFisher Scientific). The expression levels of mRNAs were calculated relative to casein diet controls, and data analyzed by the 2-∆∆CT method.^78^ IDG-SW3 cell RNA was collected and purified with the RNeasy Mini Kit (QIAGEN) according to the manufacturer’s instructions. cDNA was synthesized from 1 µg RNA with reverse transcriptase Superscript II RT (Thermo Fisher Scientific). Gene expression was analyzed by Taqman quantitative RT-PCR using custom-made primers and probes: mouse *Fgf23* (forward: 5′-AGCCAGGACCAGCTATCACCTA-3′, reverse: 5′-CTTCGAGTCATGGCTCCTGTTAT-3′). Expression levels were normalized relative to the expression of *Hprt* (forward: 5′-TTATCAGACTGAAGAGCTACTGTAATGATC-3′, reverse: 5′-TTACCAGTGTCAATTATATCT TCAACAATC-3′, probe: 5′-TGAGAGATCATCTCCACCAATAACTTTTATGTCCC-3′). For quantification of gene expression, ΔΔCt method was used.

### Statistical analysis

Data were analyzed by a one-way or two-way ANOVA where appropriate, followed by a Tukey post-hoc test or Student’s *t*-test (two-tailed). Significance for all tests was set at *P* < 0.05. Data are represented as bar or line graphs of the mean ± the standard error of the mean (SEM), or box-and-whisker plots with the middle line representing the median of the data, upper and lower quartiles within the boxes, and the whiskers as the minimum and maximum. Dots represent individual samples.

## Supplementary information

Supplmental Information

Table S9

Table S10

Table S2-S4

Table S6-S8

## Data Availability

Both RNA-seq and ATAC-seq data are deposited in GEO under accession number GSE205792.
