# Supplementary material for: Osteocyte Egln1/Phd2 links oxygen sensing and biomineralization via FGF23
Source: Bone Res. 2023 Jan 18;11:7. doi: 10.1038/s41413-022-00241-w (PMC9845350; doi:10.1038/s41413-022-00241-w)
Supplement: Supplementary file 1 — Supplmental Information [file 41413_2022_241_MOESM1_ESM.docx]

**Supplementary Information for**

Osteocyte *Egln1*/Phd2 links oxygen sensing and biomineralization via FGF23

Megan L. Noonan^1^, Pu Ni^1^, Emmanuel Solis^1^, Yamil G. Marambio^1^, Rafiou Agoro^1^, Xiaona Chu^1^, Yue Wang^1^, Hongyu Gao^1^, Xiaoling Xuei^1^, Erica L. Clinkenbeard^1^, Guanglong Jiang^1^, Sheng Liu^1^, Steve Stegen^6^, Geert Carmeliet^6^, William R. Thompson^2,3^, Yunlong Liu^1,5^, Jun Wan^1^, and Kenneth E. White^1,4^*

Departments of ^1^Medical and Molecular Genetics, ^2^Physical Therapy, ^3^Anatomy, Cell Biology, and Physiology, ^4^Medicine/Division of Nephrology, and ^5^Center for Computational Biology and Bioinformatics, Indiana University School of Medicine, Indianapolis, IN, USA, 46202. ^6^Laboratory of Clinical and Experimental Endocrinology, Department of Chronic Diseases and Metabolism, KU Leuven, 3000 Leuven, Belgium.

*Corresponding author: Kenneth E. White

Email: [kenewhit@iu.edu](mailto:kenewhit@iu.edux)

Fig. S1. Effects of HIF-PHI on human U2OS cells. (A) Transferrin receptor (*TFRC*) expression and (B) *FGF23* expression in human U2OS cells treated with 20μM or 50μM of the HIF-PHI FG (FG-4592; Roxadustat), AKB (AKB-6548; Vadadustat), or BAY (BAY 85-3934; Molidustat) for 24h (blue) or 48h (red). (*p<0.05, **p<0.01, ***p<0.001 versus vehicle; #p<0.05 24h versus 48h). (A, inset) HIF1α protein expression in U2OS cells after 4h of 20μM or 50μM HIF-PHI treatment.

Fig. S2. Principal component analysis (PCA) of (A) ATACseq and (B) RNAseq samples. (C) Volcano plots of more open regions (red) and more closed regions (blue) genome-wide in FG-4592-treated MSCs and osteocytes compared to vehicle treatment. (D) Volcano plots of upregulated (red) and downregulated (blue) genes in FG-4592-treated MSCs and osteocytes compared to vehicle treatment. (E) Expression values (in logCPM) of *Tfrc* and *Egln1* (Phd2) in RNAseq data from FG-4592 treated and vehicle-treated MSCs and osteocytes.

Fig. S3. Representative ATACseq peaks of the mouse *Fgf23* gene and -20 kb upstream of untreated and FG-4592-treated MSCs (top 2 tracks) and untreated and FG-4592-treated osteocytes (bottom 2 tracks). Highlighted regions identify peaks within and near the TSS of Fgf23 and a previously identified -16 kb enhancer region.

Fig. S4. (A) Phd2, Phd1, and Phd3 mRNA expression (1/raw Ct value) in osteocyte-enriched bone fractions of wild-type mice (***p<0.001 vs Phd2). (B) Intact FGF23 concentrations in casein diet- (CD) and adenine diet (AD)-fed mice (*p<0.05 vs CD 8wk). (C) Serum intact FGF23 concentrations in flox-Phd2/Dmp1-cre- and -cre+ mice.

**Fig. S5. (A)** Phd2 (*Egln1*) and **(B)** *Fgf23* mRNA expression in 6 undifferentiated CRISPR PHD2-KO clones compared to WT cells showing clone A2 (green arrow) with undetectable Phd2 expression and basal elevations in *Fgf23* which was used for further study. **(C)** Secreted intact FGF23 and **(D)** secreted total FGF23 in 3-week differentiated Phd2-KO cells compared to WT (p<0.01 vs WT). **(E)** *Fgf23* mRNA expression in CRISPR/Cas9-generated Phd2-KO IDG-SW3 cells (**p<0.01 vs gScr control cells).

Table S1. ATACseq number of DARs. Number of differentially accessible regions (DAR) UP (more open) or DOWN (more closed) by comparison in ATACseq samples (FDR<0.05).

| Comparison | Number of peaks UP | Number of peaks DOWN |
| --- | --- | --- |
| uFG vs uVeh | 1333 | 1312 |
| dFG vs dVeh | 453 | 219 |
| dFG vs uFG | 7224 | 5877 |
| dVeh vs uVeh | 8561 | 5658 |

Table S2. Significant differentially accessible regions (DAR) for FG-4592-treated MSC (undiff_FG) vs vehicle-treated MSC (undiff_veh) (FDR <0.05)

*See supplemental file ‘Table S2-S4 ATACseq DAR.xlsx’*

Table S3. Significant differentially accessible regions (DAR) for FG-4592-treated Ocy (3wk_FG) vs vehicle-treated Ocy (3wk_veh) (FDR <0.05)

*See supplemental file ‘Table S2-S4 ATACseq DAR.xlsx’*

Table S4. Significant differentially accessible regions (DAR) for vehicle-treated Ocy (3wk_veh) vs vehicle-treated MSC (undiff_veh) (FDR <0.05)

*See supplemental file ‘Table S2-S4 ATACseq DAR.xlsx’*

Table S5. RNAseq number of DEGs. Number of differentially expressed genes (DEG) UP or DOWN by comparison in RNAseq samples (logFC>|1|, FDR<0.05).

| Comparison | Number of genes UP | Number of genes DOWN |
| --- | --- | --- |
| uFG vs uVeh | 780 | 1006 |
| dFG vs dVeh | 1172 | 800 |
| dFG vs uFG | 2248 | 1041 |
| dVeh vs uVeh | 1764 | 1133 |

Table S6. Significant differentially expressed genes (DEG) for FG-4592-treated MSC (uFG) vs vehicle-treated MSC (uVeh) (logFC>1, FDR <0.05)

*See supplemental file ‘Table S6-S8 RNAseq DEG.xlsx’*

Table S7. Significant differentially expressed genes (DEG) for FG-4592-treated Ocy (dFG) vs vehicle-treated Ocy (dVeh) (logFC>1, FDR <0.05)

*See supplemental file ‘Table S6-S8 RNAseq DEG.xlsx’*

Table S8. Significant differentially expressed genes (DEG) for vehicle-treated Ocy (dVeh) vs vehicle-treated MSC (uVeh) (logFC>1, FDR <0.05)

*See supplemental file ‘Table S6-S8 RNAseq DEG.xlsx’*

Table S9. Significant Canonical Pathways (p<0.05) identified by IPA in FG-4592-treated MSC (uFG) vs vehicle-treated MSC (uVeh)

*See supplemental file ‘Table S9 IPA Canonical Pathways uFG vs uVeh.xlsx’*

Table S10. Significant Canonical Pathways (p<0.05) identified by IPA in FG-4592-treated Ocy (dFG) vs vehicle-treated Ocy (dVeh)

*See supplemental file ‘Table S10 IPA Canonical Pathways dFG vs dVeh.xlsx’*

Table S11. Top 3 most significant genes in more open ATACseq (DAR) regions and upregulated in RNAseq (DEG) for FG-treated versus vehicle-treated MSCs and osteocytes (Ocy), and in Ocy vs MSC (vehicle-treated).
